# Construction of a prognostic model based on eight ubiquitination-related genes *via* machine learning and potential therapeutics analysis for cervical cancer

**DOI:** 10.3389/fgene.2023.1142938

**Published:** 2023-03-14

**Authors:** Yiping Hao, Mutangala Muloye Guy, Qingqing Liu, Ruowen Li, Zhonghao Mao, Nan Jiang, Bingyu Wang, Baoxia Cui, Wenjing Zhang

**Affiliations:** Department of Obstetrics and Gynecology, Qilu Hospital of Shandong University, Jinan, China

**Keywords:** cervical cancer, ubiquitination-related genes, bioinformatics, prognosis model, potential therapeutics, machine learning

## Abstract

**Introduction:** Ubiquitination is involved in many biological processes and its predictive value for prognosis in cervical cancer is still unclear.

**Methods:** To further explore the predictive value of the ubiquitination-related genes we obtained URGs from the Ubiquitin and Ubiquitin-like Conjugation Database, analyzed datasets from The Cancer Genome Atlas and Gene Expression Omnibus databases, and then selected differentially expressed ubiquitination-related genes between normal and cancer tissues. Then, DURGs significantly associated with overall survival were selected through univariate Cox regression. Machine learning was further used to select the DURGs. Then, we constructed and validated a reliable prognostic gene signature by multivariate analysis. In addition, we predicted the substrate proteins of the signature genes and did a functional analysis to further understand the molecular biology mechanisms. The study provided new guidelines for evaluating cervical cancer prognosis and also suggested new directions for drug development.

**Results:** By analyzing 1,390 URGs in GEO and TCGA databases, we obtained 175 DURGs. Our results showed 19 DURGs were related to prognosis. Finally, eight DURGs were identified *via* machine learning to construct the first ubiquitination prognostic gene signature. Patients were stratified into high-risk and low-risk groups and the prognosis was worse in the high-risk group. In addition, these gene protein levels were mostly consistent with their transcript level. According to the functional analysis of substrate proteins, the signature genes may be involved in cancer development through the transcription factor activity and the classical P53 pathway ubiquitination-related signaling pathways. Additionally, 71 small molecular compounds were identified as potential drugs.

**Conclusion:** We systematically studied the influence of ubiquitination-related genes on prognosis in cervical cancer, established a prognostic model through a machine learning algorithm, and verified it. Also, our study provides a new treatment strategy for cervical cancer.

## 1 Introduction

Among cancers in females, cervical cancer incidence and mortality are high (Siegel et al., 2021; Sung et al., 2021). Because of the increasing implementation of systematic screening and the introduction of the HPV vaccine, its incidence was partly decreased. However, the incidence remains high in places with poor economic levels (Zhao and Qiao, 2019). In addition, most patients were in late stages when diagnosed, and the prognosis was poor. Therefore, prognostic biomarkers still need to be investigated to distinguish high-risk patients for personalized treatment and follow-up strategy.

Ubiquitination, a post-translational modification, regulates protein function or degradation (Nakamura, 2018). In eukaryotes, the process of protein ubiquitination is a three-step thioester cascade process involving enzymes including E1s (ubiquitin-activating enzymes), E2s (ubiquitin-conjugating enzymes), and E3s (ubiquitin-protein ligases) (Zheng and Shabek, 2017). The E1 enzyme activates the 76-amino acid ubiquitin followed by transferring activated ubiquitin to the E2 enzyme. Finally, E3 is responsible for recruiting a specific substrate and catalyzing ubiquitin transfer from E2 to the protein (Song and Luo, 2019). Among the seven lysine residues and one methionine residue that comprise ubiquitin, each has the ability to bind another ubiquitin moiety, producing a protein that is either monoubiquitinated or polyubiquitinated, making it a highly versatile and elaborate post-translational modification (Weissman, 2001). In addition, ubiquitin on substrate proteins can be removed by deubiquitinating enzymes (DUBs), leading to a reverse process of ubiquitination (Komander et al., 2009). One of the most well-known functions of ubiquitination is to facilitate protein degradation (Muratani and Tansey, 2003). Since ubiquitination targets a wide range of substrates, it contributes to most intracellular molecular biological processes, regulating tumor progression, and mediating therapeutic resistance as well (Hoeller and Dikic, 2009; Huang and Dixit, 2016).

Ubiquitination is attracting increasing attention, and several studies proved ubiquitination involved cervical cancer. Martin et al. demonstrated that HPV E6 protein promoted p53 degradation by ubiquitin-dependent proteinases (Scheffner et al., 1990). Then, further study revealed that E6 binds to ubiquitin-ligase E6AP, promoting the development of cervical cancer by degrading p53 (Martinez-Zapien et al., 2016). Huh et al. reported that HPV16 E7-associated cullin 2–ubiquitin ligase complex contributes to the aberrant degradation of the pRB tumor suppressor (Huh et al., 2007). In addition, ubiquitination proteins are potentially promising targets for cancer therapy (Wang et al., 2021; Yang et al., 2021). Morgan et al. (2021) demonstrated that USP13 deubiquitinates and stabilizes Mcl-1, promoting the proliferation in cervical cancer. Additionally, they found that BH3 mimetic inhibitor, a USP13 inhibitor, could induce cell death by reducing Mcl-1 expression. Yi et al. (2020) found UBE2L3 caused excessive p53 ubiquitination by nuclear export of HP1γ. They also found that doxorubicin promoted HP1γ-mediated UBE2L3 inhibition, increasing p53 stability and activity in cisplatin-resistant cervical cancer cells. With its extensive substrates and the ability to regulate protein levels, the ubiquitination pathway has become a promising therapeutic route (Huang and Dixit, 2016). Therefore, systematic analysis of ubiquitination-related genes and construction of a ubiquitination-related gene signature to predict prognosis in cervical cancer is undoubtedly necessary.

In this study, we obtained ubiquitination-related genes by searching the Ubiquitin and Ubiquitin-like Conjugation Database (IUUCD) and analyzed the dataset from TCGA and GEO databases. We identified several ubiquitination-related genes associated with prognosis significantly, including RBBP4, SRM, GCH1, USP14, TRAIP, CBX4, VEZF1, and TOM1. Also, these ubiquitination-related genes were used to develop a reliable prognostic signature. The ubiquitination-related prognostic signature was used to differentiate patients into two groups, with the high-risk groups having worse outcomes. Therefore, our signature can help doctors to establish personalized treatment and follow-up plans according to risk stratification. In addition, to find a mechanism for how ubiquitin proteasome regulates cervical cancer, we predicted the substrate proteins and did the functional analysis. Through the CMap database, we identified 71 small molecular compounds identified as potential compounds, and they were involved in 34 mechanisms, including the inhibitor of actin polymerization, AKT, ALK, aurora kinase, CDK, dehydrogenase, DNA-dependent protein kinase, FLT3, focal adhesion kinase, glucosyltransferase, DNA protein kinase, HDAC, HMGCR, IGF-1, EGFR, IKK, JNK, MEK, RAF, VEGFR, MTOR, PI3K, protein kinase, protein synthesis, DNA synthesis, topoisomerase, tyrosine kinase, Coflilin signaling pathway activator, estrogen receptor antagonist, glucokinase activator, HIF modulator, mitochondrial oxidative phosphorylation uncoupler, retinoid receptor ligand, and T-type calcium channel blocker. The study provides new guidelines for evaluating cervical cancer outcomes and suggests new directions for drug development.

## 2 Methods

### 2.1 Obtained and processed datasets

Gene expression data, along with clinical information, were collected from the NCBI Gene Expression Omnibus (GEO) database^1^ and The Cancer Genome Atlas (TCGA) database^2^. Since these are public databases, this study is exempted from ethical review and does not require patients to sign informed consent. The selection criterion required the dataset to have relevant clinical information. Finally, GSE39001, GSE52903, GSE44001, and TCGA-CESC datasets were enrolled for analysis (Table 1).

**TABLE 1:** Overview of details of the datasets.

GEO accession	Platform		Total	Normal	Cancer	Clinical outcome
GSE39001	GPL201	[HG-Focus] Affymetrix Human HG-Focus Target Array	55	12	43	OS
	GPL6244	[HuGene-1_0-st] Affymetrix Human Gene 1.0 ST Array [transcript (gene) version]	24	5	19	
GSE52903	GPL6244	[HuGene-1_0-st] Affymetrix Human Gene 1.0 ST Array [transcript (gene) version]	72	17	55	OS
GSE44001	GPL14951	Illumina HumanHT-12 WG-DASL V4.0 R2 expression beadchip	300	0	300	DFS
TCGA-CESC		Illumina	309	3	306	OS

OS, overall survival and DFS, disease-free survival.

The GSE39001 data include data from two sequencing platforms, GPL201 and GPL6244, in which the GPL201 platform includes 12 normal samples and 43 cancer samples, and the GPL6244 platform included five normal samples and 19 cancer samples (Espinosa et al., 2013). GSE52903 was derived from the GPL6244 platform and included 17 normal samples and 55 cancer samples (Medina-Martinez et al., 2014). For data from different platforms, we only combined data from the same company to reduce the batch effect. Since both GSE39001 and GSE52903 are Affymetrix-sequencing companies, so we integrated the two cohorts to increase the sample size. Then, a metadata cohort was created and used to identify genes differentially expressed between cancer and normal samples. In both prognostic studies and further mechanistic studies, we used study cohorts with GSE52903 and GSE39001 integration.

As a unified standardized process, Affymetrix microarray datasets were preprocessed and normalized using the RMA function in the Affy package including background correction and normalization (Irizarry et al., 2003). Moreover, batch effects were removed using the SVA package’s combat function (Leek, 2014). Following the batch effect removal, normalization was performed using the normalizeBetweenArrays method. Principal component analysis (PCA) is used to extract principal components and can be used to distinguish the distribution of samples by the prcomp function using the ggbiplot package.

TCGA RNA sequencing data (TPM format) were downloaded from the Genomic Data Commons^2^. In addition, for the GSE44001 dataset from the Illumina company containing 300 samples, the clinical outcome was disease-free survival (DFS), and the signature accuracy in predicting disease progression was examined (Lee et al., 2013). The TCGA-CESC cohort is also from the Illumina platform, and we used the TCGA-CESC cohort as a validation cohort to explore the signature accuracy. We also downloaded annotation files from NCBI GEO for various microarray platforms. Gene symbols were generated based on the probe annotation files for each dataset. The probe average was calculated for gene symbols with more than one probe.

The integrated annotations for the Ubiquitin and Ubiquitin-like Conjugation Database contains E1s, E2s, E3s, DUBs, UBDs, and ULDs for 74 families of all 68 animals, 39 plants, and 41 fungal species. In addition, the IUUCD provides annotation information for all these proteins in the database. We downloaded the human ubiquitination-related genes from IUUCD and organized them. In total, 1,390 ubiquitination-related genes (URGs) were obtained from the IUUCD^3^ (Gao et al., 2013; Zhou et al., 2018).

To clearly show the process of this study, we drew a flow chart as shown in Supplementary Figure S1A.

### 2.2 Screened DURGs

We identified differentially expressed genes (DEGs) between tumors and normal samples by the R package limma. Our screening criterion was adjusted *p*-values <0.001. R package VennDiagram was used to intersect DEGs with URGs to identify differentially expressed URGs. Plotting of ubiquitination-related DEGs (DURGs) expression data in all samples was performed by the pheatmap package.

### 2.3 Functional analysis and visualization

We conducted Gene Ontology (GO) and Kyoto Encyclopedia of Genes and Genomes (KEGG) enrichment for DURGs by the clusterProfiler package (Yu et al., 2012). The visualization and localization of KEGG pathways were performed based on DURGs by the pathview packages.

On the KEGG pathway legend, genes are categorized according to their differential significance and increase or decrease, in which red indicates upregulation and green indicates a decrease. In addition, the color shades correspond to the value of log_10_ [fold change]. Significant pathways had *p*-value <0.05.

### 2.4 Established and validated the prognostic gene signature

We identified DURGs that have an association with the overall survival through univariate Cox regression in metadata data. After the filtration of DURGs associated with overall survival, candidate DURGs were selected *via* integrated analysis of two machine learning algorithms consisting of the Least Absolute Shrinkage and Selection Operator (LASSO) algorithm with penalty parameter tuning conducted by 10-fold cross-validation and the Support Vector Machine-Recursive Feature Elimination (SVM-RFE) algorithm searching for lambda with the smallest classification error to determine the variable (Duan et al., 2005).

We performed LASSO regression using the R package glmnet. Furthermore, SVM-RFE was performed by msvmRFE.R^4^ based on the e1071 R package. Next, we merged the results of LASSO and SVM-RFE to identify the top-ranking common genes. Following the aforementioned filtration process, prognostic gene signatures were constructed by multivariate Cox regression. Also, we calculated patients’ risk scores, the cutoff value of which was ensured by the function of surv_cutpoint in the survminer package. Then, two groups of patients were categorized as high- and low-risk. In addition, we further explored the prognostic performance of each gene in the prognostic gene signature for cervical cancer.

Additionally, the prognostic gene signature was validated in TCGA cohorts. To further explore the predictive effect of the gene signature on tumor progression, we used GSE44001 for verification. For TCGA and GSE44001, risk scores were calculated in the same way, and the same method was used to group patients. The Kaplan–Meier analysis with log-rank test and univariate and multivariate Cox regression was performed by the survival package and the ROC curve was constructed by the survivalROC package. From the ROC curve, we also calculated the area under the ROC curve (AUC). The value of AUC ranges between 0.5 and 1. The closer the AUC is to 1.0, the higher the authenticity. If it is equal to 0.5, the authenticity is the lowest and has no application value.

In addition, we also further explore the role of the risk score calculated from the gene signature in the diagnosis of cervical cancer by logistic analysis using the rms package.

### 2.5 Analysis of signature genes protein expression in the HPA database

The Human Protein Atlas^5^ (HPA) offers open access to data for exploring the proteome of humans and has helped many academicians. We used the HPA database to detect the protein levels of signature genes by immunohistochemistry (IHC), and we obtained IHC images from the database (Uhlen et al., 2015).

### 2.6 Predicted substrate proteins and performed functional enrichment analysis

It has been known that E3s and DUBs bind specifically to substrates among these enzymes. UbiBrowser^6^ was used to predict the substrate protein of E3s and DUBs (Li et al., 2017; Wang et al., 2022). Afterward, we selected 20 substrate proteins with the highest prediction scores and known substrate proteins for functional enrichment analysis by the clusterProfiler package.

### 2.7 Identified potential drugs by connectivity map analysis

The Connectivity Map^7^ (CMap) is a chemical genomics database, and its resource can help researchers identify relationships between small molecules, diseases, and drugs (LAMB et al., 2006). We used the query function in the CMap to identify potential drugs. According to guidelines, up- and downregulated DURGs were uploaded on the online tools. In general, a negative enrichment value relates to a drug’s potential for treating the disease, with a greater value indicating more efficacy. Compounds identified by the CMap were filtered based on enrichment scores (ES, Score < −90).

### 2.8 Statistical analysis

Statistical analyses are performed by R version 4.1.3. All analyses were two-sided with *p* < 0.05 considered statistically significant.

## 3 Results

### 3.1 Grouped samples and identified prognostic DURGs

**FIGURE 1:**
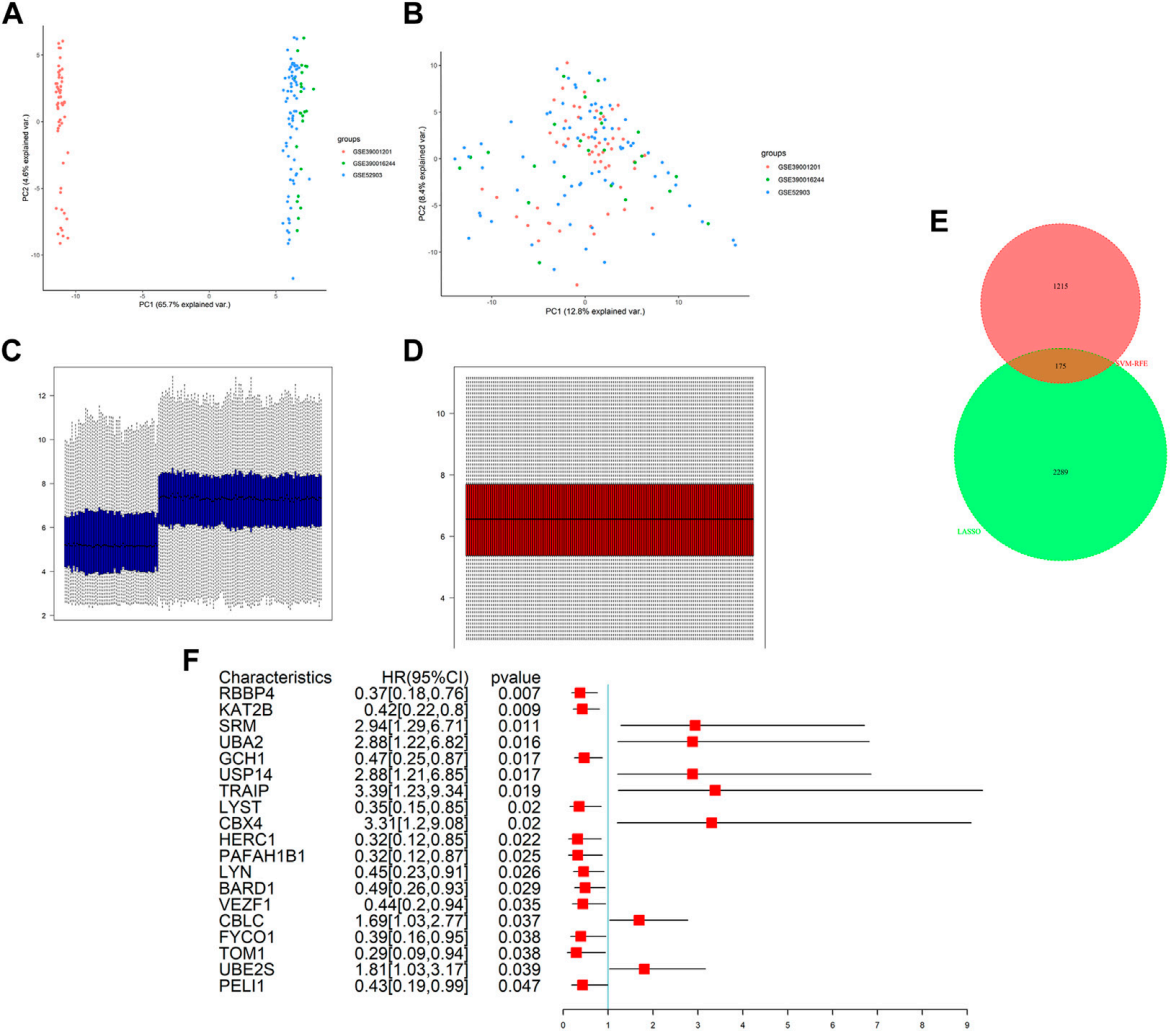
Data preprocessing and differential expression analysis. PCA analysis before **(A)** and after **(B)** batch effect adjustment and normalization. Barplot before **(C)** and after **(D)** batch effect adjustment and normalization. **(E)** Intersection analysis of DEGs and ubiquitination-related genes. **(F)** Results of univariate Cox regression analysis of DURGs.

We integrated two datasets, namely, GSE52903 and GSE39001, into a comprehensive dataset for investigating gene expression in cervical cancer after removing the batch effects by sva package and normalizing by normalizeBetweenArrays methods. Following this, the distributions of the samples were determined by PCA before and after correction. Figures 1A,C present the distributions of the original datasets, while Figures 1B,D show the removal of confounding factors. From Figure 1, we can see that before removing the batch effect, the samples of each dataset are clustered together, and after removing the batch effect, the samples are evenly distributed.

After integration, the total number of gene probes was 7,824. Additionally, differential expression analysis revealed 2,464 DEGs in the metadata, including 1,268 upregulated genes and 1,196 downregulated genes. In addition, expression levels were also shown visually in Supplementary Figure S2. In total, 1,390 URGs were found in the IUUCD .

We obtained 175 DURGs from a cross-section of DEGs and URGs (Figure 1E). Among the 175 DURGs, upregulations totaled 108 and downregulations totaled 67. In total, 19 DURGs, RBBP4, KAT2B, SRM, UBA2, GCH1, USP14, TRAIP, LYST, CBX4, HERC1, PAFAH1B1, LYN, BARD1, VEZF1, CBLC, FYCO1, TOM1, UBE2S, and PELI1, were associated with OS based on univariate Cox regression analysis, with seven genes associated with poor outcomes and 12 genes associated with good outcomes (Figure 1F).

### 3.2 Functional enrichment analysis and ubiquitination mechanism assessment by KEGG map

We performed functional enrichment analysis on these 175 DURGs. According to GO analysis, DURGs were primarily involved in ubiquitin-dependent protein catabolic processes, protein polyubiquitination, and regulation of protein ubiquitination regulation. Based on KEGG analysis, DURG functions were involved in ubiquitin-mediated proteolysis, Fc gamma R-mediated phagocytosis, proteasome, and NF-kappa B signaling pathway. Based on KEGG and GO enrichment analysis, we found that genes were mainly enriched in ubiquitination-related pathways, so we visualized using the KEGG annotation map. From this, we recognized the DURGs in the ubiquitination pathway and their effect on cancer progression (Supplementary Figure S1B).

### 3.3 Constructed gene signature to predict prognosis with eight hub DURGs

**FIGURE 2:**
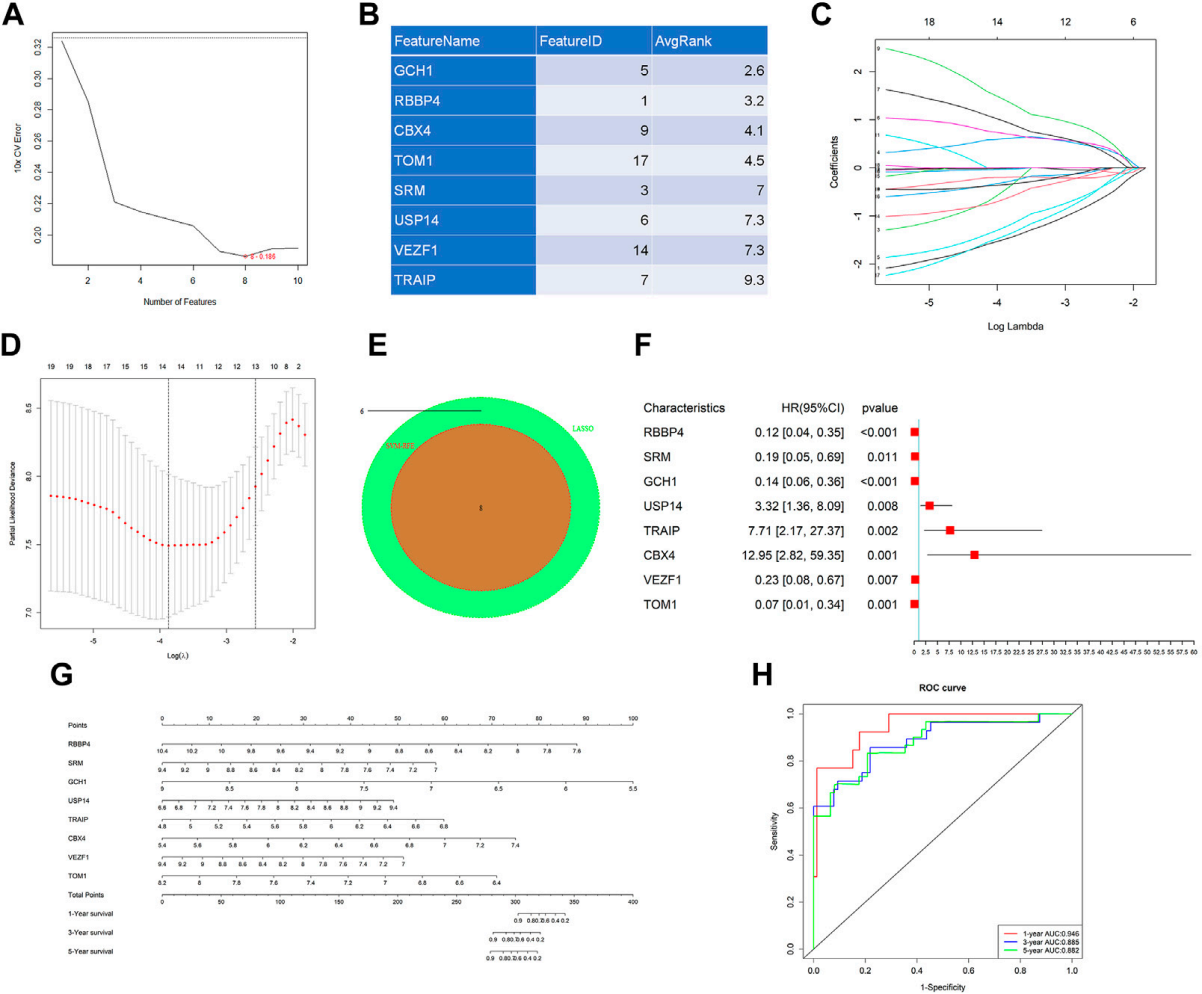
Machine learning analysis and establishment of a prognostic model. Two algorithms were used for feature selection: SVM-RFE **(A and B)** and LASSO **(C and D)** algorithms. **(E)** Intersection of two algorithms. **(F)** Forest plot of multivariate Cox regression analysis by eight hub genes. **(G)** Nomogram of the gene signature for predicting patient survival. **(H)** ROC curves of the gene signature on 1-, 3-, and 5-year OS in the metadata.

Considering that too many candidate genes were identified by univariate Cox regressions, we used LASSO regression and SVM-RFE to ensure the genes we selected were important in the development of the disease. SVM-RFE analysis first identified the candidate genes. Eight genes, which were RBBP4, SRM, GCH1, USP14, TRAIP, CBX4, VEZF1, and TOM1, were identified (Figures 2A,B). In addition, LASSO analysis also identified 14 key genes, RBBP4, SRM, UBA2, GCH1, USP14, TRAIP, LYST, CBX4, HERC1, BARD1, VEZF1, FYCO1, TOM1, and PELI1, from 19 genes (Figures 2C,D). The intersection of LASSO and SVM-RFE analyses revealed eight hub genes in cervical cancer, which were RBBP4, SRM, GCH1, USP14, TRAIP, CBX4, VEZF1, and TOM1 (Figure 2E). Eventually, we performed multivariate Cox analysis on eight hub genes to construct the gene signature to predict the prognosis (Figure 2F). Based on the coefficients of each gene, the risk score was calculated as follows: risk score = (−2.1476*RBBP4)+(−1.6532*SRM)+(−1.9507*GCH1)+(1.1985*USP14)+(2.0422*TRAIP)+(2.5609*CBX4)+(−1.4570*VEZF1) +(−2.6949*TOM1). Additionally, the gene signature was visualized by using a nomogram (Figure 2G). The gene signature’s AUC at 1, 3, and 5 years were 0.946, 0.885, and 0.882, respectively, indicating the model had high accuracy and reliability (Figure 2H).

In addition, we further explored the prognostic value of each gene in the gene signature, and the results were shown in Supplementary Figure S3. The results showed that the tags were more predictive of the prognosis than any one of the genes.

### 3.4 Prognostic gene signature validated in GEO datasets and TCGA cohort

**FIGURE 3:**
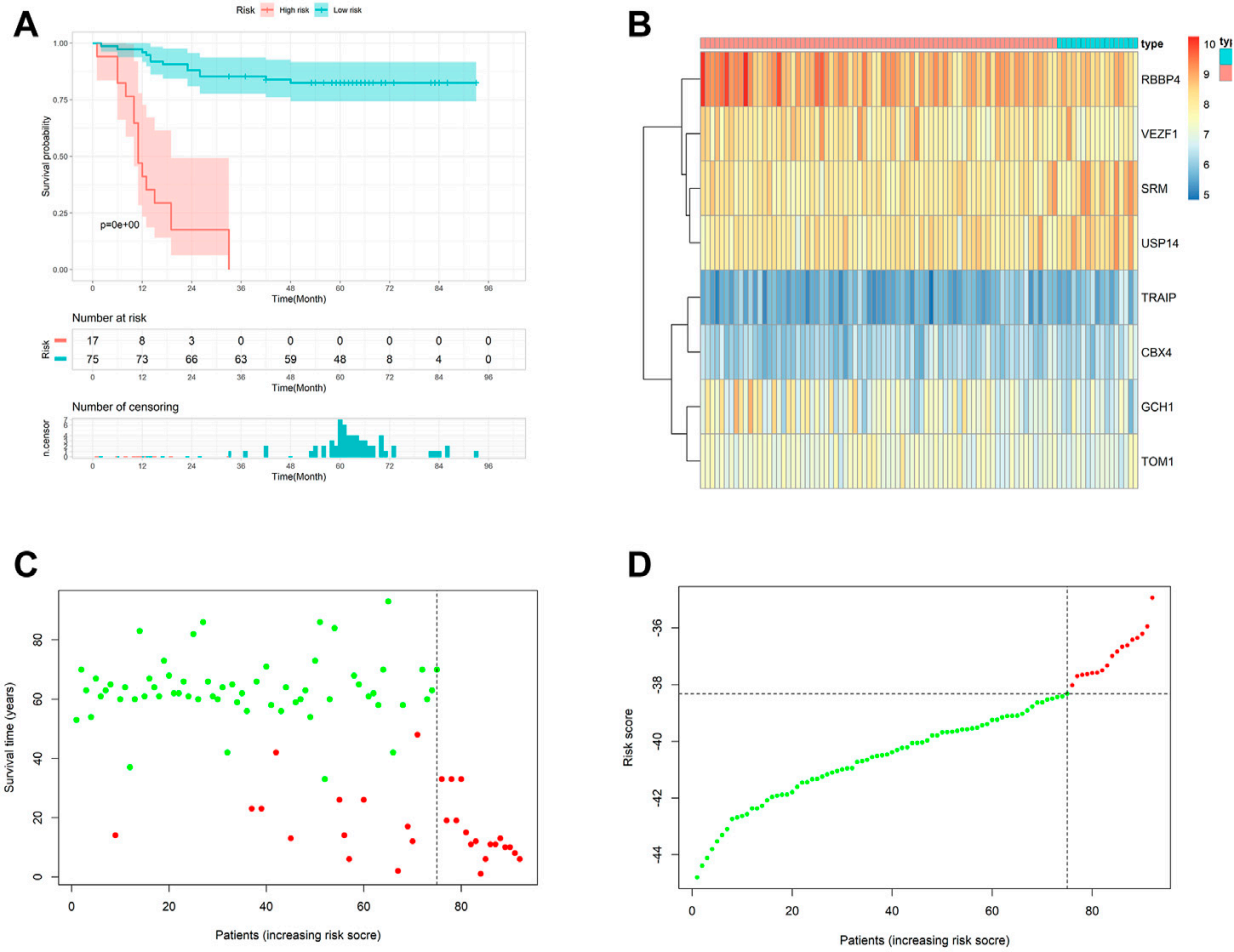
Internal validation of the prognostic model. **(A)** Kaplan–Meier curves with log-rank test in the metadata. **(B)** Heatmap of the hub genes between low- and high-risk groups in the metadata. **(C and D)** Risk scores distribution of the metadata.

We calculated the risk scores in the metadata and based on the cutoff points calculated, using the survminer package, and we classified all patients into high- and low-risk groups (Figures 3C,D). Patients with high risk in the metadata cohort had worse outcomes than patients with low risk, according to Kaplan–Meier log-rank analysis (Figure 3A). Figure 3B showed the mRNA expression level of eight signature genes in the metadata.

We calculated the risk scores in TCGA-CESC and grouped patients as mentioned previously to validate the gene signature reliability (Supplementary Figure S4D, E). As shown in Supplementary Figure S4A, high-risk patients also had significantly worse prognoses in TCGA cohort. Additionally, the heatmap showed the landscape of the eight signature genes in TCGA-CESC cohort (Supplementary Figure S4B). In addition, the ROC curve also proved the conclusion (Supplementary Figure S4C).

In the GSE44001 cohort, the outcome variable was disease-free time. To investigate whether gene signature plays a role in disease progression, we used GSE4401 to explore further. We also calculated the risk scores and grouped patients as mentioned previously (Supplementary Figure S5D, E). We found that disease-free survival time was shorter in a high-risk group (Supplementary Figure S5A). Additionally, Supplementary Figure S5B showed eight signature gene expressions in GSE44001.

The AUC of 1, 3, and 5 years DFS were 0.621,0.610, and 0.588, respectively, all over 0.5, indicating that they had a certain reference value in predicting disease progression (Supplementary Figure S5C). It is further suggested that our gene signature had good performance on the occurrence and development of cervical cancer and may be indispensable in cervical cancer.

### 3.5 Constructed a nomogram by risk scores and clinical factors

**FIGURE 4:**
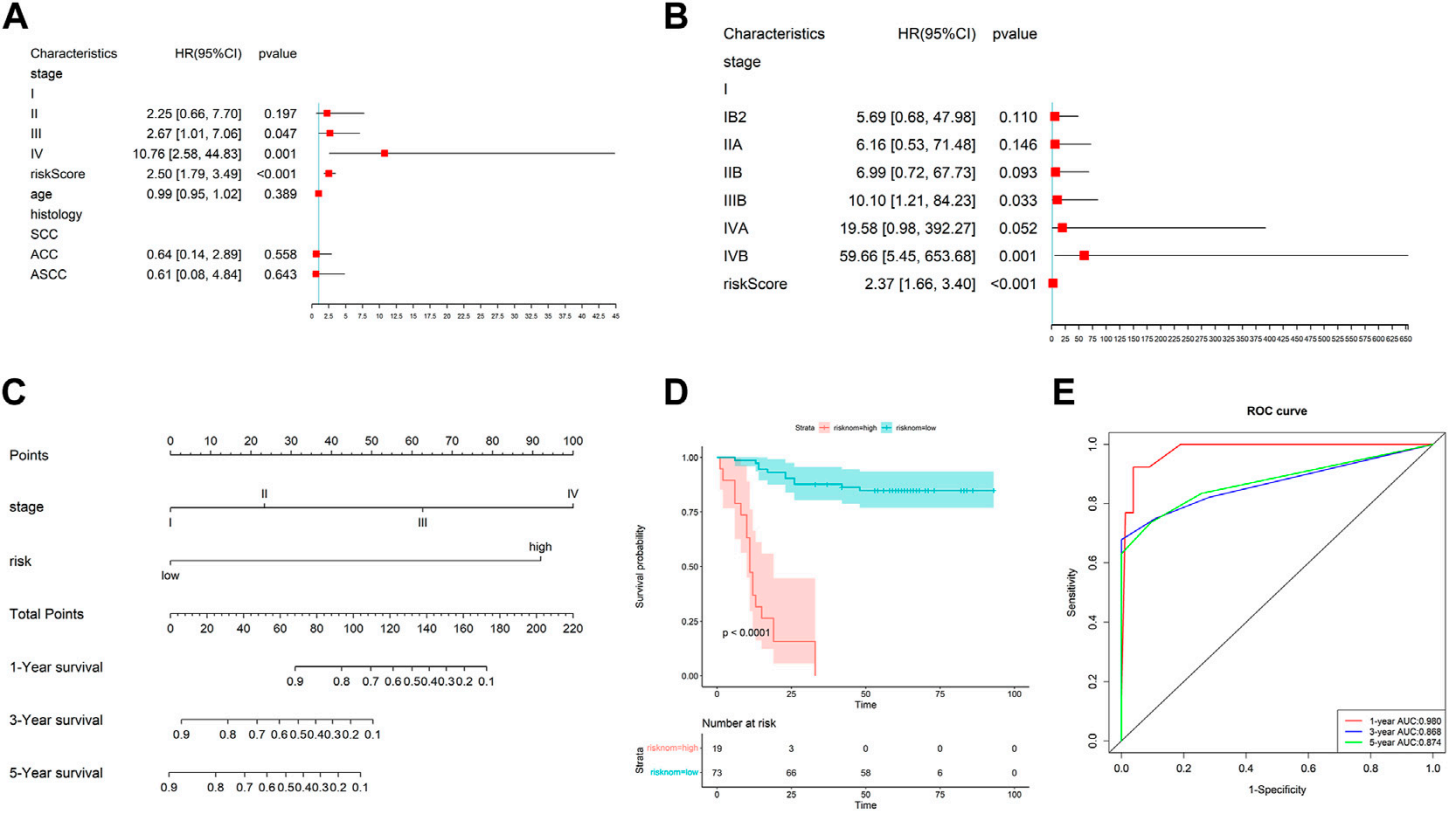
Effect of prognostic models and clinicopathological factors on survival. **(A)** Univariate Cox analysis of risk scores and other clinical features. **(B)** Multivariate Cox analysis showed the risk score was an independent prognostic factor. **(C)** Nomogram constructed by the risk score and clinical factors. **(D)** K–M analysis of the nomogram. **(E)** ROC curves of the nomogram on 1-, 3-, and 5-year OS.

A systematic analysis was performed based on their risk scores generated by the gene signature and clinical characteristics, such as stage and age to explore the gene signature relationship with clinical factors. Figure 4A shows the risk score was closely correlated with OS. Also, the risk score was an independent prognostic factor demonstrated by multivariate Cox regression analysis (Figure 4B). Moreover, we studied the risk scores between different histological types and different stages, as shown in Supplementary Figure S6B-C. The results showed that there was no significant difference in the risk scores among different histological types. However, the later the clinical stage, the higher the risk score. We further explored the value of the risk score calculated by the gene signature in cervical cancer diagnosis. The AUC was 0.679 (Supplementary Figure S6A). The result indicated that the gene signature was also valuable in the diagnosis of cervical cancer.

Then, we took the risk score as a variable to construct the other nomogram to predict the patient’s OS (Figure 4C). The results showed that the nomogram has an excellent performance on the predicted 1-year OS. Also, the nomogram also can divide the patients clearly (Figure 4D). The AUC of the nomogram for 1-, 3-, and 5-year OS were 0.980, 0.868, and 0.874, respectively (Figure 4E).

**FIGURE 5:**
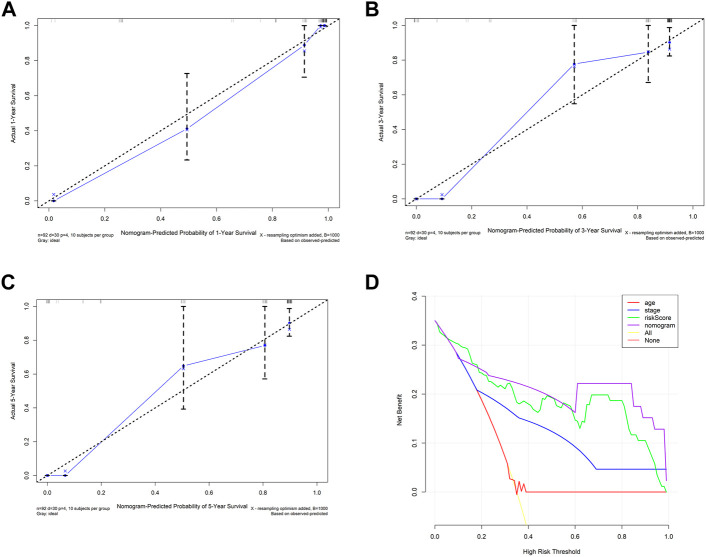
Internal validation of prognostic models with clinicopathological factors. **(A–C)** Calibration curves of the nomogram constructed by the risk score and clinical factor. **(D)** Decision curve of the nomogram constructed by the risk score and clinical factor.

Calibrate curves showed the comparison between the predicted probability and the observed probability of 1-, 3-, and 5-year OS. In addition, almost perfect calibration curves were observed in Figures 5A–C. To compare the predictive effect of the nomogram with the risk score and other clinical factors for predicting the prognosis, we further constructed decision curves. The results showed that the nomogram calculated by the risk score and clinical factors has the best performance (Figure 5D).

### 3.6 Validation of the protein levels of the signature genes

**FIGURE 6:**
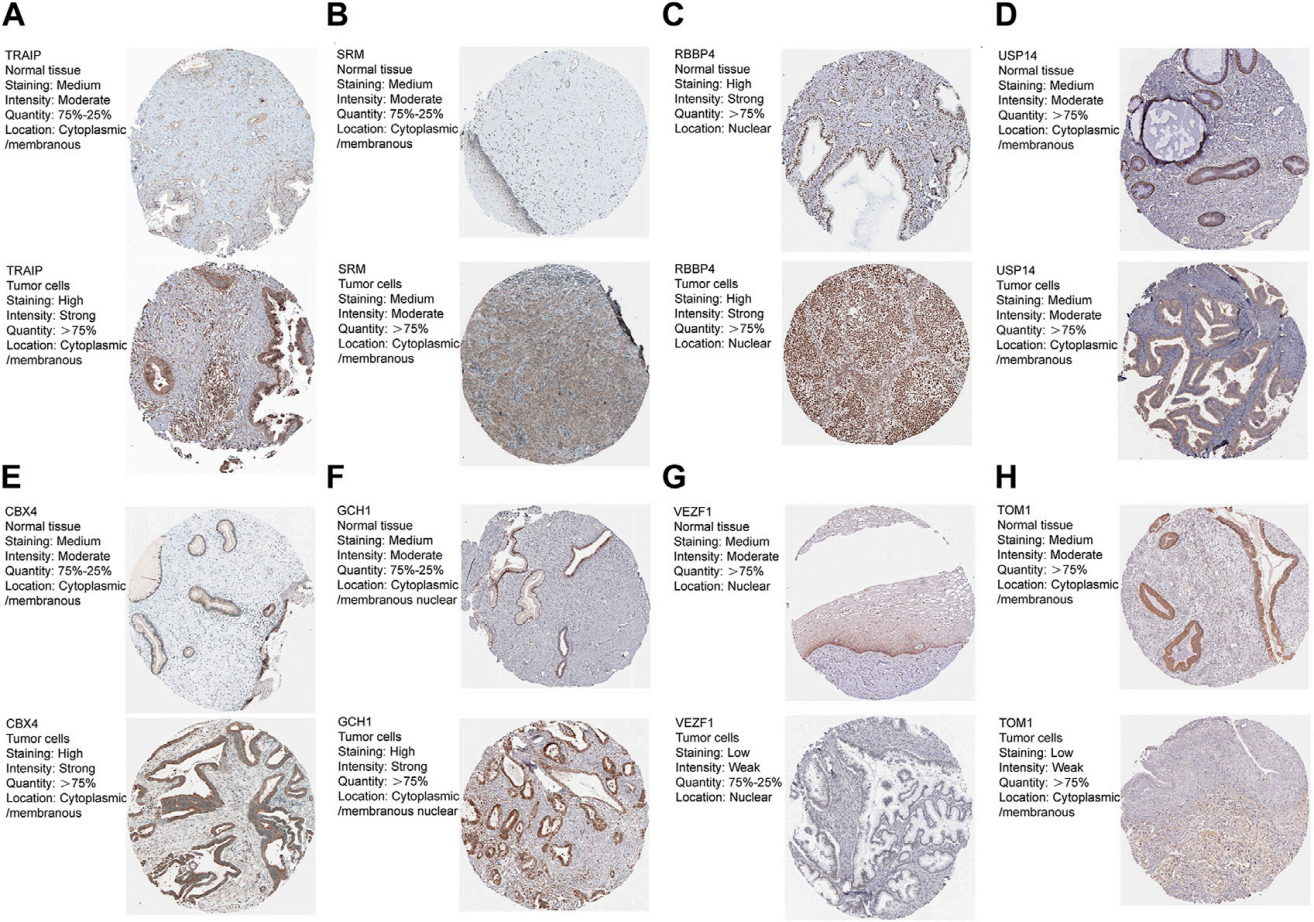
**(A-H)** Eight genes protein levels between normal and cancer tissues from the HPA database.

We obtained immunohistochemistry staining from the HPA database to further explore the signature genes’ protein levels. The results are shown in Figures 6A–H. As mentioned previously, the mRNA expression level of eight signature genes in the metadata are shown in Figure 3B. Among these genes, GCH1, USP14, CBX4, TRAIP, VEZF1, and TOM1 protein levels were consistent with the transcript. Tumors and normal groups both had high RBBP4 protein levels, and SRM protein levels were moderate in both normal and tumor groups.

### 3.7 Detection of the substrate proteins for E3s and DUBs in the gene signature and functional enrichment analysis

**FIGURE 7:**
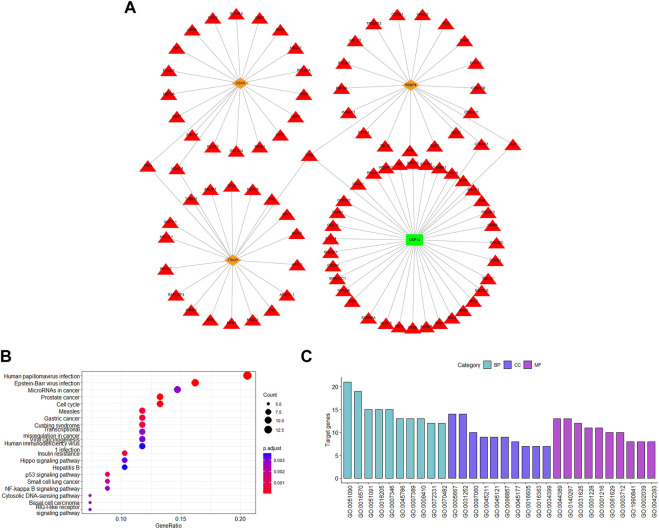
Analysis of substrates for ubiquitination-related genes. **(A)** Top 20 predicted and known substrate proteins. **(B)** KEGG enrichment analysis. **(C)** GO pathway enrichment analysis. The red triangle represents the substrate proteins; the orange diamond represents the E3s; and the green rectangle represents the DUB.

UbiBrowser was used to predict the possible substrate proteins for the signature genes to determine the potential functional impact. Among the eight genes, RBBP4, CBX4, and TRAIP are E3s and USP14 is DUBs. SRM is a predicted E3 and has not been experimentally confirmed to have substrate proteins. The number of the predicted substrate proteins of RBBP4, CBX4, and TRAIP was 71, 110, and 317, respectively. In addition, TRAIP has one known substrate protein. USP14 is a DUB that has 18 known substrate proteins and 382 predicted substrates proteins. Further functional analysis was carried out on the top 20 predicted substrates (Figure 7A).

According to GO analysis, substrate proteins participate in the regulation of transcription factor activity, histone modification, protein modification, regulating the classical P53 pathway, and ubiquitination-related signaling pathways (Figure 7C). Additionally, according to KEGG analysis, the substrate proteins were associated with the human papillomavirus infection pathway, which also confirmed the gene signature validity. In addition, the substrate proteins also participated in some intriguing pathways, such as cell cycle, p53 signaling pathway, cytosolic DNA-sensing pathway, and NF-kappa B signaling pathway (Figure 7B). Our signature genes are involved in these pathways, illustrating that they may promote cervical cancer development through these pathways. Also, it helps us to identify the potential therapeutic drugs.

### 3.8 Identified small molecular compounds for cervical cancer

In addition, we screened the DURGs for potential therapeutic drugs for cervical cancer using the CMap database. There were 71 small molecular compounds identified as potential compounds, and they were involved in 34 mechanisms, including the inhibitor of actin polymerization, AKT, ALK, aurora kinase, CDK, dehydrogenase, DNA dependent protein kinase, FLT3, focal adhesion kinase, glucosyltransferase, DNA protein kinase, HDAC, HMGCR, IGF-1, EGFR, IKK, JNK, MEK, RAF, VEGFR, MTOR, PI3K, protein kinase, protein synthesis, DNA synthesis, topoisomerase, tyrosine kinase, Coflilin signaling pathway activator, estrogen receptor antagonist, glucokinase activator, HIF modulator, mitochondrial oxidative phosphorylation uncoupler, retinoid receptor ligand, and T-type calcium channel blocker (Table 2).

**TABLE 2:** Results of CMap analysis.

ID	Name	Description	Score
BRD-K67868012	PI-103	MTOR inhibitor	−99.26
BRD-K12184916	Dactolisib	MTOR inhibitor	−99.12
BRD-K67566344	KU-0063794	MTOR inhibitor	−99.05
BRD-K69932463	AZD-8055	MTOR inhibitor	−98.94
BRD-K30677119	PP-30	RAF inhibitor	−98.89
BRD-K63068307	ZSTK-474	PI3K inhibitor	−98.76
BRD-K77008974	WYE-354	MTOR inhibitor	−98.26
BRD-K64606589	Apicidin	HDAC inhibitor	−98.1
BRD-K52911425	GDC-0941	PI3K inhibitor	−98.1
BRD-K97365803	PI-828	PI3K inhibitor	−98.08
BRD-K13049116	BMS-754807	IGF-1 inhibitor	−97.92
BRD-K04887706	AKT-inhibitor-1-2	AKT inhibitor	−97.88
BRD-A62025033	Temsirolimus	MTOR inhibitor	−97.82
BRD-K79090631	CGP-60474	CDK inhibitor	−97.71
BRD-K68065987	MK-2206	AKT inhibitor	−97.71
BRD-A11678676	Wortmannin	PI3K inhibitor	−97.6
BRD-K99545815	PF-562271	Focal adhesion kinase inhibitor	−97.5
BRD-K92428153	Mycophenolate-mofetil	Dehydrogenase inhibitor	−97.45
BRD-K99818283	PIK-90	PI3K inhibitor	−97.43
BRD-K27305650	LY-294002	MTOR inhibitor	−97.43
BRD-K94294671	OSI-027	MTOR inhibitor	−97.29
BRD-K08589866	Linsitinib	IGF-1 inhibitor	−97.19
BRD-K00337317	NU-7441	DNA-dependent protein kinase inhibitor	−97.04
BRD-K12502280	TG-101348	FLT3 inhibitor	−96.9
BRD-U51951544	ZG-10	JNK inhibitor	−96.71
BRD-K34581968	BMS-536924	IGF-1 inhibitor	−96.6
BRD-K40175214	Torin-1	MTOR inhibitor	−96.58
BRD-K09499853	KU-0060648	DNA-dependent protein kinase inhibitor	−96.51
BRD-K06750613	GSK-1059615	PI3K inhibitor	−96.46
BRD-K64800655	PHA-793887	CDK inhibitor	−96.41
BRD-A45498368	WYE-125132	MTOR inhibitor	−96.41
BRD-K06792661	Narciclasine	Coflilin signaling pathway activator	−96.37
BRD-K77908580	Entinostat	HDAC inhibitor	−96.31
BRD-K23192422	Lestaurtinib	FLT3 inhibitor	−96.13
BRD-U82589721	HG-5-113-01	Protein kinase inhibitor	−96.02
BRD-K56334280	Amonafide	Topoisomerase inhibitor	−95.95
BRD-M16762496	PIK-75	DNA protein kinase inhibitor	−95.87
BRD-K04210847	Tamoxifen	Estrogen receptor antagonist	−95.74
BRD-K91370081	Anisomycin	DNA synthesis inhibitor	−95.67
BRD-K07762753	Aminopurvalanol-a	Tyrosine kinase inhibitor	−95.65
BRD-K51575138	TPCA-1	IKK inhibitor	−95.36
BRD-K53414658	Tivozanib	VEGFR inhibitor	−95.21
BRD-K68174511	Torin-2	MTOR inhibitor	−94.82
BRD-K69840642	ISOX	HDAC inhibitor	−94.79
BRD-K80348542	Cephaeline	Protein synthesis inhibitor	−94.79
BRD-K89626439	Sirolimus	MTOR inhibitor	−94.77
BRD-K05653692	DL-PDMP	Glucosyltransferase inhibitor	−94.17
BRD-K09549677	Mibefradil	T-type calcium channel blocker	−94.08
BRD-K49294207	BIBU-1361	EGFR inhibitor	−94.08
BRD-K29733039	Deforolimus	MTOR inhibitor	−93.96
BRD-K78431006	Crizotinib	ALK inhibitor	−93.91
BRD-K58772419	AZD-6482	PI3K inhibitor	−93.86
BRD-K06543683	Bisindolylmaleimide-ix	CDK inhibitor	−93.76
BRD-K76674262	Homoharringtonine	Protein synthesis inhibitor	−93.62
BRD-K41895714	AS-605240	PI3K inhibitor	−93.58
BRD-K68488863	ENMD-2076	FLT3 inhibitor	−93.26
BRD-M64432851	Sunitinib	FLT3 inhibitor	−93.24
BRD-A25687296	Emetine	Protein synthesis inhibitor	−93.23
BRD-K36740062	GSK-1070916	Aurora kinase inhibitor	−93.18
BRD-A81772229	Simvastatin	HMGCR inhibitor	−92.24
BRD-K14821540	FCCP	Mitochondrial oxidative phosphorylation uncoupler	−92.11
BRD-K75295174	Alisertib	Aurora kinase inhibitor	−92.1
BRD-U44700465	HG-5-88-01	Protein kinase inhibitor	−91.34
BRD-A26002865	Verrucarin-a	Protein synthesis inhibitor	−91.3
BRD-K56343971	Vemurafenib	RAF inhibitor	−91.27
BRD-K57080016	Selumetinib	MEK inhibitor	−91.23
BRD-A19248578	Latrunculin-b	Actin polymerization inhibitor	−90.8
BRD-K13927029	Retinol	Retinoid receptor ligand	−90.73
BRD-K21672174	RO-28-1675	Glucokinase activator	−90.43
BRD-K68336408	Tyrphostin-AG-1478	EGFR inhibitor	−90.06
BRD-K44432556	VU-0418946-1	HIF modulator	−90

## 4 Discussion

It has been reported that protein ubiquitination regulates the growth or death of tumor cells through various biological processes by changing the ubiquitination level of the substrate protein, inducing the degradation or stabilization of the substrate protein (Wang et al., 2019). Further research of these URGs will help broaden our horizons in cervical cancer development and prognosis of cervical cancer patients. Through bioinformatics methods, a few previous studies have been focused on cervical cancer prognosis. Pan et al. screened the m6A RNA methylation regulator genes and constructed a prognostic signature (Pan et al., 2020). Jiang et al. identified the autophagy-related gene and constructed a prognostic model (Jiang et al., 2021). Until now, no bioinformatics study has been conducted on the ubiquitination of cervical cancer. Therefore, we focused on protein ubiquitination to develop a prognostic model.

Cervical cancer URGs were systematically investigated. By analyzing 1,390 URGs in the GEO and TCGA databases, we obtained 175 DURGs. Also, 19 DURGs were related to OS among these DEGs. Then, we screened eight hub DURGs by SVM-RFE and LASSO regression analysis, and then the eight hub DURGs had multivariate Cox regression performed to construct the prognostic model. The model’s AUC were 0.946, 0.885, and 0.882 at 1-, 3-, and 5- years OS, which indicated that it can accurately predict the prognosis of patients. Next, the risk scores were calculated and classified patients into high- and low-risk groups. Based on the results, cervical cancer patients with different survival outcomes could be accurately separated. We can formulate treatment plans and follow-up strategies according to different risk stratifications. In addition, the model’s reliability and stability were further validated in TCGA-CESC cohort, and it could also accurately predict the prognosis and divide patients into two groups with different prognoses, which indicated that the prognostic gene signature was stable. We further integrated the clinical factors into analysis including age, histology, and stage and multivariate Cox analysis revealed the risk score was an independent prognostic factor.

Among these genes in the prognostic gene signature, it has been confirmed that RBBP4 could control HPV16 transforming activity in cervical cancer. When overexpressed, it inhibited cell growth and tumor formation significantly (Kong et al., 2007). Also, RBBP4 was also associated with radiosensitivity. Zheng et al. found that RBBP4 could enhance radiosensitivity *in vivo* and *in vitro* (Zheng et al., 2013). As for USP14, Xu et al. found that USP14 could stabilize MDM2 (Xu et al., 2020). MDM2 could mediate p53 ubiquitination and induce p53 degradation (Hock and Vousden, 2014). Furthermore, USP14 selective inhibitor IU1 decreased MDM2 expression, inhibited growth, and triggered apoptosis in cervical cancer cells (Xu et al., 2020). We did not find other gene-associated studies in cervical cancer. TRAIP enhances osteosarcoma invasion and proliferation through the modulation of IGFBP3/AKT by promoting the degradation of KANK1, which is a tumor suppressor (Li et al., 2021). Zhu et al. found that the overexpression of SRM induced chemotherapy resistance in bladder cancer cells (Zhu et al., 2022). CBX4 promotes proliferation through affecting BMI-1 expression in lung cancer cells (Hu et al., 2020). In hepatocellular carcinoma, GCH1 silencing promotes cell growth by activating superoxide anion-mediated ASK1/p38 signaling (Zhong et al., 2021). VEZF1 and TOM1 have been investigated in few studies. Then, we investigate the protein levels of the eight genes in the HPA database. Overall, protein expression levels for most genes were consistent with their transcriptional levels. It appears that the genes we identified are worthy of further investigation.

We performed a functional enrichment analysis of 175 DURGs between normal and cancerous tissues. According to GO analysis, DURGs were mainly involved in ubiquitin-dependent proteolytic metabolic processes, protein polyubiquitination, and regulation of protein ubiquitination. KEGG analysis revealed that DURGs were functionally involved in ubiquitin-mediated proteolysis, Fc γ r-mediated phagocytosis, and proteasome and NF-kappa B signaling pathways. In addition, among the eight genes, RBBP4, CBX4, TRAIP, and USP14 specifically bind to substrate proteins. According to the functional analysis of substrate proteins, substrate proteins are involved in transcription factor regulation, histone modification, protein modification, and other related pathways, regulation of the classical p53 pathway, and regulation of the mitotic cell cycle and ubiquitination-related signaling pathways. The common pathways involved in ubiquitination genes and their substrates are mainly involved in protein modification or degradation, such as the ubiquitinated proteasome system. Among them, ubiquitination modifications mainly involve protein degradation, such as Fc γ r-mediated phagocytosis, while substrates are mainly involved in protein modifications, such as histone modifications. We suggest that these genes influence prognosis through these pathways.

As with any study, ours has some limitations as well. Our study was retrospective and lacked prospective clinical trial validation. Further experimental studies are needed to confirm the mechanism of DURGs. Therefore, we will collect clinical specimens in the near future and conduct basic experiments to further verify our results.

In conclusion, the prognostic gene signature based on the ubiquitination of cervical cancer was first constructed and validated. In addition, it can accurately predict patients’ OS. Through this gene signature, we can distinguish high- and low-risk groups, so as to formulate individualized treatment plans and follow-up strategies. In addition, the risk score calculated by the gene signature was also an independent prognostic factor. Additionally, we identified the TFs and substrate protein associated with the prognostic signature genes to gain a deeper understanding of their underlying molecular biological mechanisms. In addition, we also conducted drug predictions through DURGs and obtained 71 small molecule compounds, which may inhibit the occurrence and development of cervical cancer. Furthermore, these eight genes may serve as new biomarkers or targets for cervical cancer research.

## Data Availability

The datasets presented in this study can be found in online repositories. The names of the repository/repositories and accession number(s) can be found in the article/Supplementary Material.

## Footnotes

1
https://www.ncbi.nlm.nih.gov/geo/.

2
https://portal.gdc.cancer.gov/.

3
http://iuucd.biocuckoo.org/.

4
https://github.com/johncolby/SVM-RFE.

5
http://www.proteinatlas.org.

6
http://ubibrowser.ncpsb.org.

7
https://clue.io/.
