# Supplementary figures and images for: Construction of a prognostic model based on eight ubiquitination-related genes via machine learning and potential therapeutics analysis for cervical cancer
Source: Front Genet. 2023 Mar 14;14:1142938. doi: 10.3389/fgene.2023.1142938 (PMC10043205; doi:10.3389/fgene.2023.1142938)

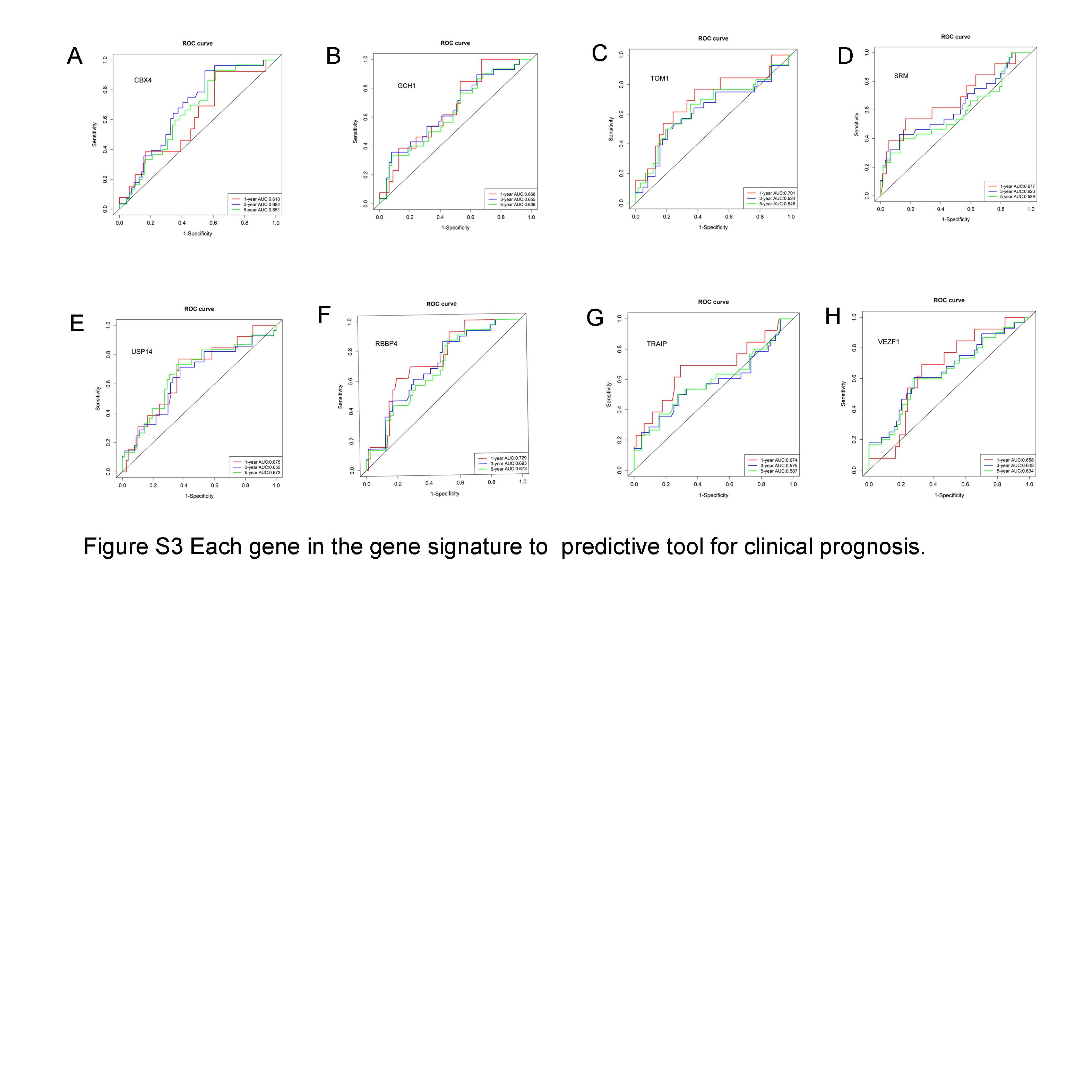

Supplement: Supplementary file 1 [file Image3.JPEG]

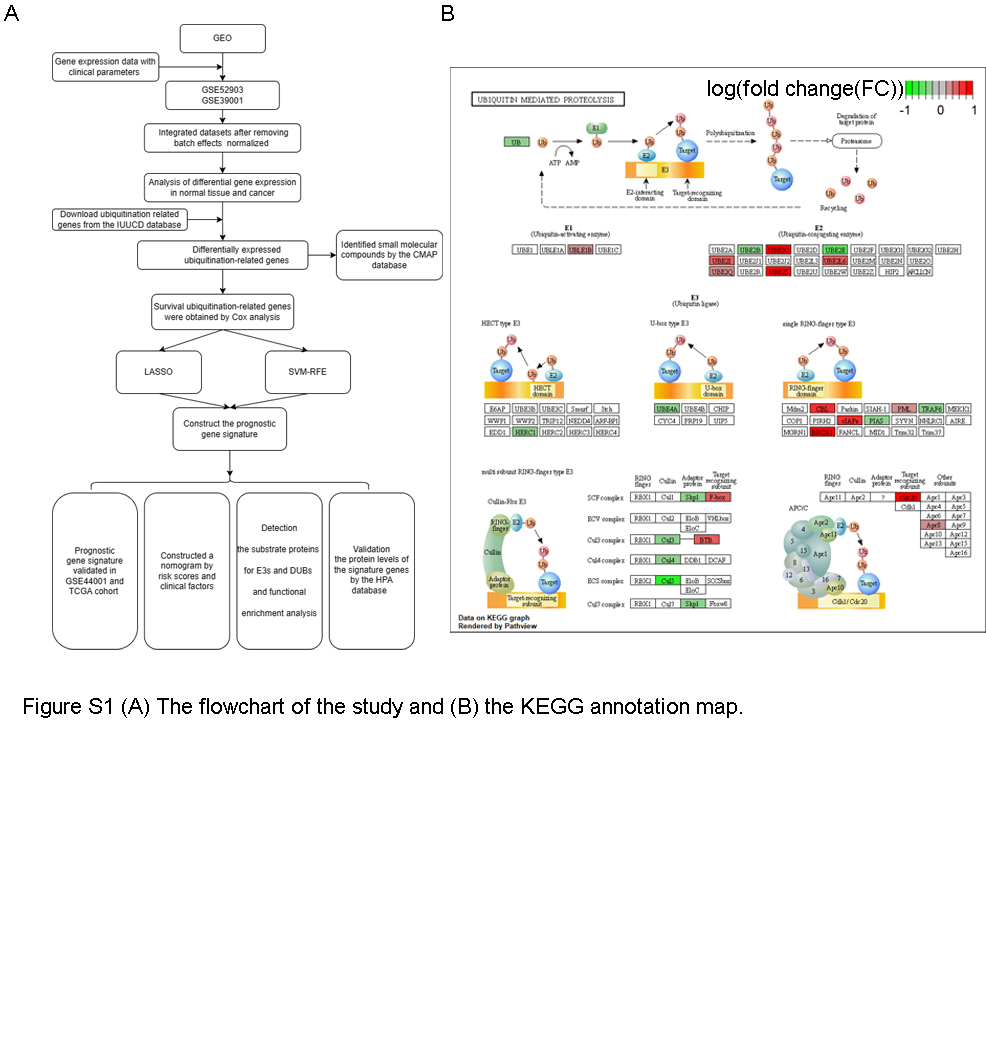

Supplement: Supplementary file 2 [file Image1.JPEG]

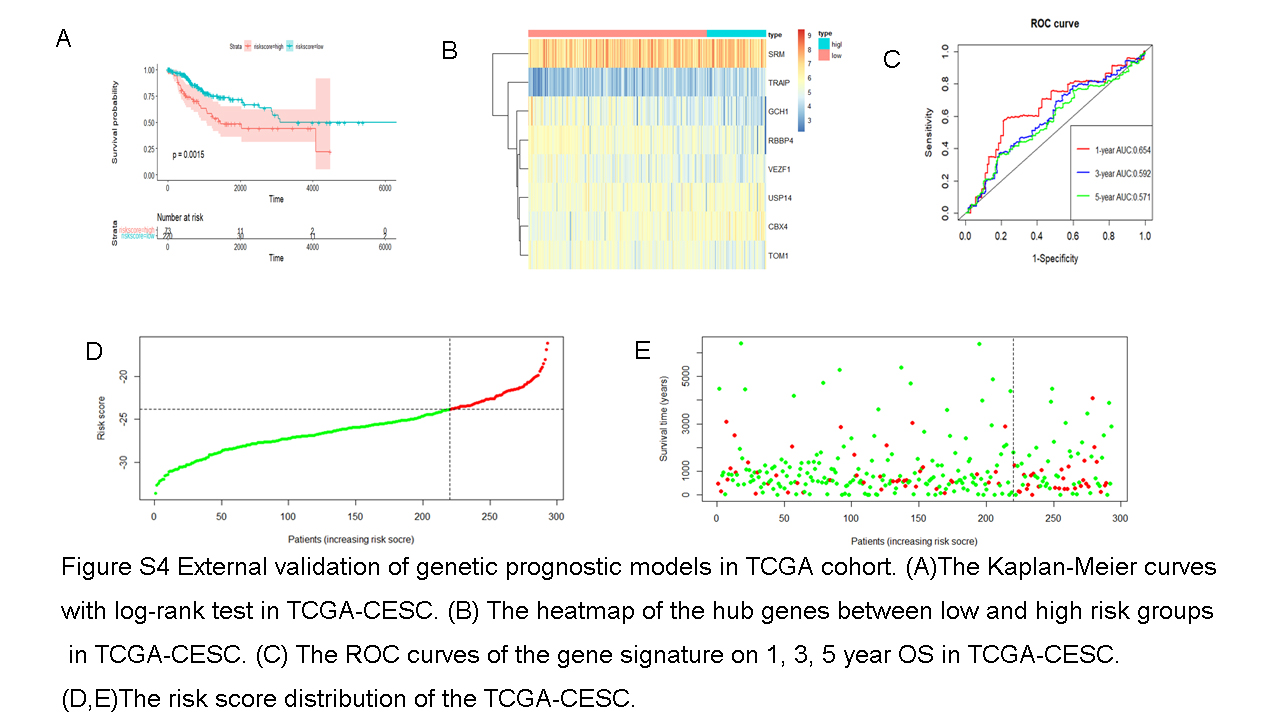

Supplement: Supplementary file 3 [file Image4.JPEG]

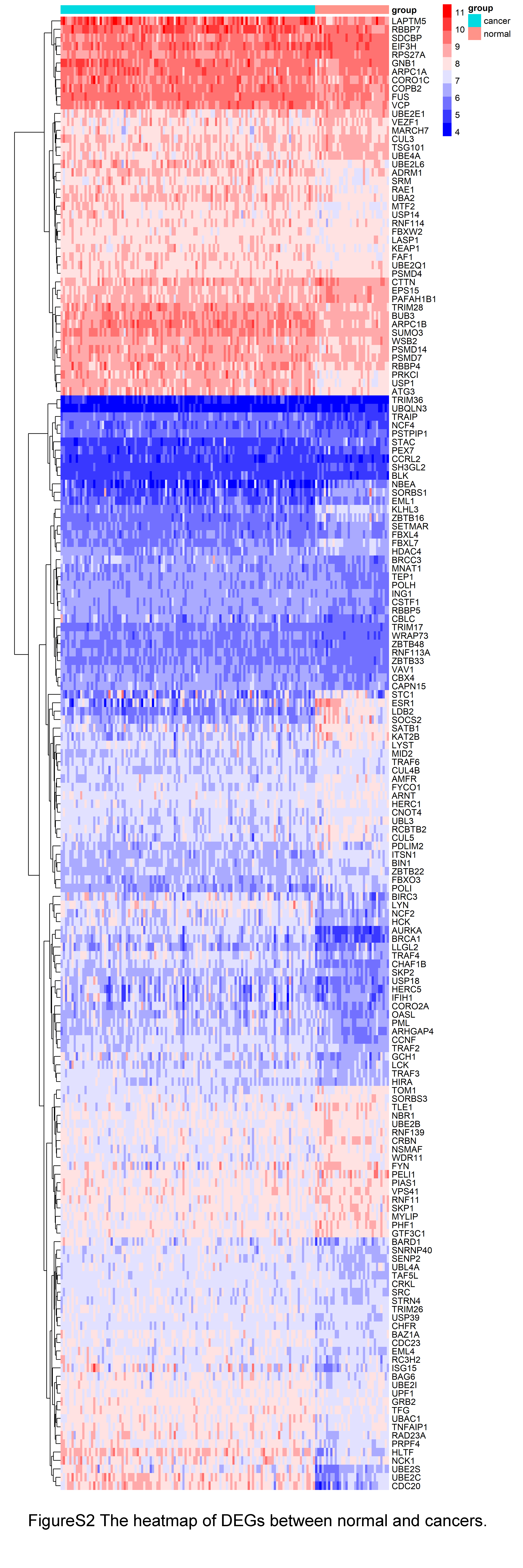

Supplement: Supplementary file 4 [file Image2.JPEG]

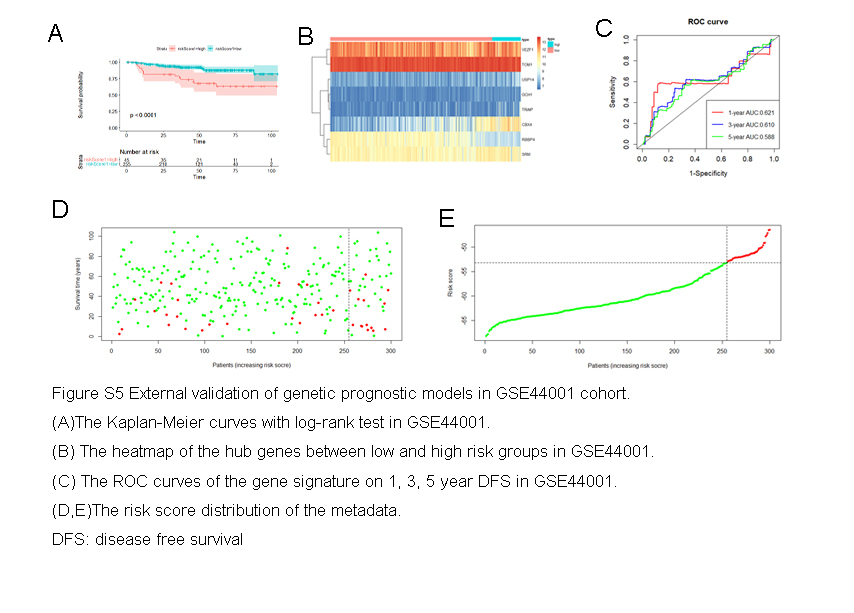

Supplement: Supplementary file 5 [file Image5.JPEG]

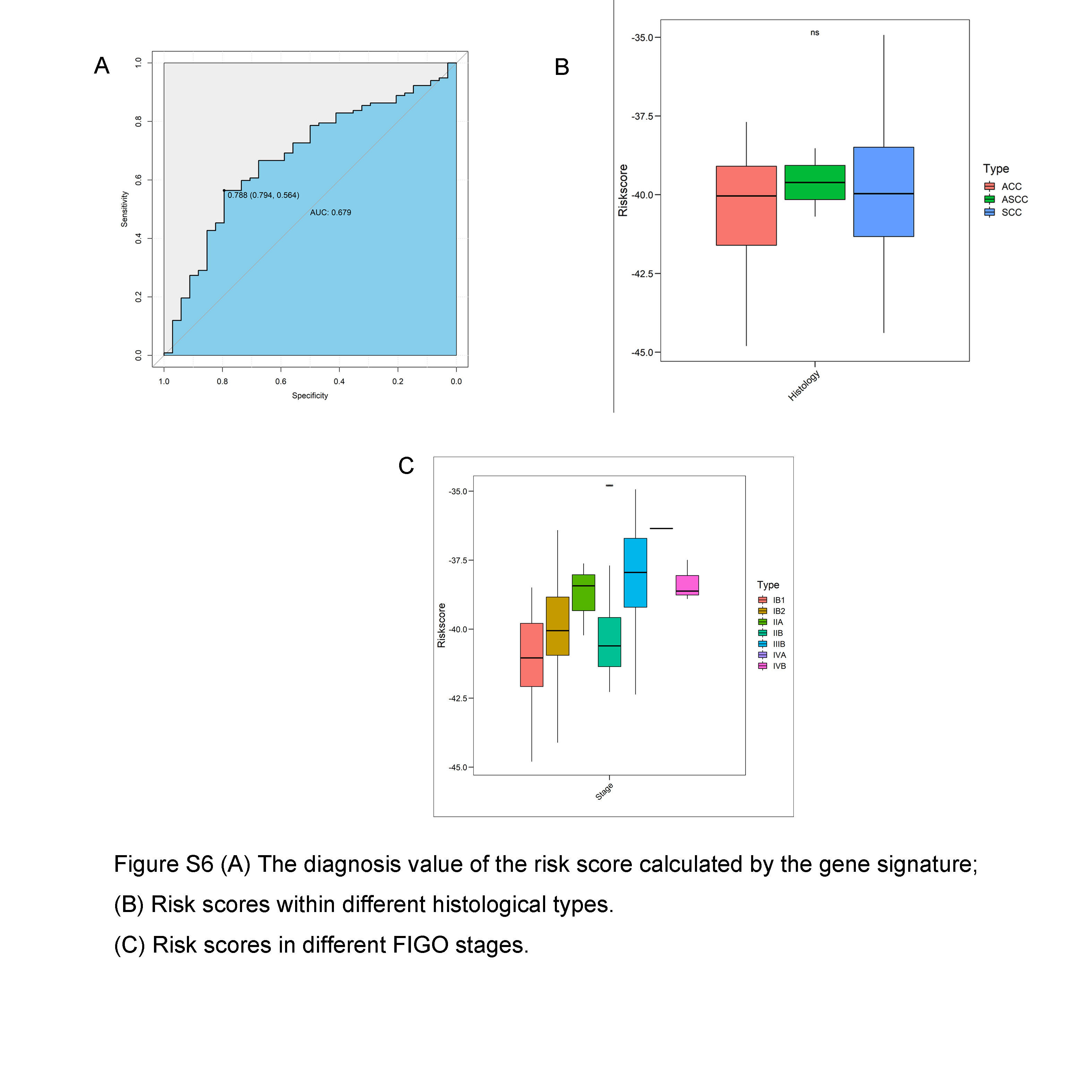

Supplement: Supplementary file 6 [file Image6.JPEG]
